# Gradient elevation of serum CYFRA21-1 and its synergy with KL-6 for risk stratification in rheumatoid arthritis-associated interstitial lung disease

**DOI:** 10.3389/fmed.2026.1763928

**Published:** 2026-04-02

**Authors:** Jingjing Yin, Sasa Liu, Na Zhao, Jun Guo, Jingyuan Wang, Wei Wei

**Affiliations:** 1 Department of Clinical Laboratory, The First Affiliated Hospital of Xi’an Jiaotong University, Xi’an, Shaanxi, China; 2 Department of Medical Informatics, The First Affiliated Hospital of Xi’an Jiaotong University, Xi’an, Shaanxi, China; 3 Department of Ultrasound Medicine, The First Affiliated Hospital of Xi’an Jiaotong University, Xi’an, Shaanxi, China

**Keywords:** cytokeratin 19 fragment antigen 21-1, interstitial lung disease, Krebs von den Lungen-6, rheumatoid arthritis, tumor markers

## Abstract

**Objective:**

This study aimed to assess the clinical value of cytokeratin 19 fragment (CYFRA21-1) and its correlation with Krebs von den Lungen-6 (KL-6) in stratifying the severity of rheumatoid arthritis-associated interstitial lung disease (RA-ILD).

**Methods:**

Data were retrospectively collected from 178 patients with rheumatoid arthritis (RA) admitted to our hospital between November 2024 and September 2025. The classification of patients into subgroups was based on chest high-resolution computed tomography (HRCT) findings, with 82 without interstitial lung disease (ILD), 53 with mild ILD, and 43 with advanced ILD. Serum KL-6 levels were measured using a latex agglutination assay, and the levels of tumor markers were determined by chemiluminescent immunoassay.

**Results:**

Serum CYFRA21-1 levels were significantly higher in both the RA-mild ILD and RA-advanced ILD groups compared with the RA-no ILD group (2.10 ng/mL vs. 1.90 ng/mL, *q* = 0.024; 3.70 ng/mL vs. 1.90 ng/mL, *q* < 0.001). Multivariate analysis demonstrated that CYFRA21-1 was an independent risk factor for RA-mild ILD [odds ratio (OR) = 1.711, 95% confidence interval (CI) = 1.160–2.523, *p* = 0.007] and RA-advanced ILD (OR = 4.819, 95% CI = 2.942–7.892, *p* < 0.001). KL-6 and CYFRA21-1 showed strong negative correlations with predicted forced vital capacity (FVC% pred), with correlation coefficients decreasing to −0.877 and −0.763 in the advanced ILD group, their positive correlation progressively increased with disease severity (*r* = 0.453–0.649). Furthermore, receiver operating characteristic (ROC) curve analysis indicated that CYFRA21-1 had diagnostic value for both RA-ILD (mild + advanced) and RA-advanced ILD. The area under the ROC curve (AUC) of CYFRA21-1 in RA-ILD was 0.73 (*p* < 0.001; sensitivity = 61.86%; specificity = 78.05%), while that in RA-advanced ILD was 0.88 (*p* < 0.001; sensitivity = 84.09%; specificity = 78.05%).

**Conclusion:**

CYFRA21-1 exhibited graded expression and a positive correlation with KL-6, serving as a valuable serum biomarker for risk stratification and severity assessment in RA-ILD.

## Introduction

As a systemic inflammatory disease, rheumatoid arthritis (RA) has a prevalence rate of approximately 0.5–1% in the general population (1, 2). Up to half of RA patients develop extra-articular manifestations, with pulmonary involvement being one of the primary causes of poor prognosis (3, 4). Interstitial lung disease (ILD) is the most common pulmonary complication of RA, and high-resolution computed tomography (HRCT) findings reveal that 60% of RA patients have ILD (5). The median survival of RA patients with ILD is significantly shorter than that of patients without ILD. While overall mortality from RA has declined, mortality attributable to rheumatoid arthritis-associated interstitial lung disease (RA-ILD) continues to rise (6, 7). Currently, an urgent need exists for biomarkers enabling early diagnosis and disease monitoring in RA-ILD.

Tumor markers correlate strongly with the severity of RA-ILD. For instance, elevated carbohydrate antigen (CA) 125 levels confer a six-fold increased risk of ILD development in RA patients (8, 9). Cytokeratin 19 fragment (CYFRA21-1), released during epithelial breakdown via apoptosis or epithelial-mesenchymal transition (EMT) (10, 11), is associated with ILD pathological features, and alveolar epithelial cell (AEC) injury is a core pathological characteristic of RA-ILD (12–14). KL-6, a MUC1 family member reflecting AEC damage and repair, is an established diagnostic and prognostic biomarker for RA-ILD (15, 16). Although both KL-6 and CYFRA21-1 reflect AEC injury, their synergistic value in RA-ILD severity stratification remains underexplored.

Accordingly, this study aimed to evaluate the expression of KL-6, CYFRA21-1, and other tumor markers in RA-ILD, investigate their correlations, and construct a combined model to provide a novel clinical tool for the risk stratification and severity assessment of RA-ILD.

## Materials and methods

### Study population

We reviewed 543 patients diagnosed with RA according to the 1987 American College of Rheumatology (ACR) and/or the 2010 ACR/European League Against Rheumatism (EULAR) classification criteria who were recruited from the Department of Rheumatology and Immunology at the First Affiliated Hospital of Xi’an Jiaotong University between November 2024 and September 2025 (17, 18). Patients were excluded if they had other autoimmune diseases, other pulmonary diseases, incomplete clinical data, or a current or prior history of malignancy. Malignancy exclusion was confirmed by a comprehensive review of medical records, including imaging, pathology reports, and oncology consultations. During the study, patients with unexplained weight loss, hemoptysis, or imaging suggestive of malignancy underwent further diagnostic evaluation, and those with confirmed malignancy were excluded. Ultimately, 178 eligible patients were enrolled, along with 90 healthy controls (HCs). This study adhered to the principles of the Declaration of Helsinki and was approved by the Clinical Research Ethics Committee of the First Affiliated Hospital of Xi’an Jiaotong University (XJTU1AF2024LSYY-624).

### HRCT-based ILD severity staging

Abnormalities on chest HRCT that reflect the severity of ILD were categorized into three groups: advanced ILD (bilateral pulmonary fibrosis involving multiple lobes, complicated by honeycombing primarily distributed in the subpleural regions and traction bronchiectasis), mild ILD (changes affecting > 5% of any lobar region, including non-dependent reticular abnormalities, ground-glass opacities, non-emphysematous cysts, traction bronchiectasis, honeycombing or diffuse centrilobular nodularity), and no-ILD (absence of ILD-related abnormalities, including ground-glass opacities, reticular shadows, nodular shadows, fibrosis, honeycombing, or traction bronchiectasis) (19–21). Visual mild/advanced staging was used as the primary subgrouping criterion, and semi-quantitative scoring was employed for quantitative severity verification (mild ILD: total score 3–8 points; advanced ILD: total score ≥ 9 points). This study only included patients with clearly defined mild or advanced ILD classifications (22, 23).

The extent of ILD was assessed using the following standardized method: three HRCT images were selected, corresponding to the carina level, aortic arch level, and level 1 cm above the diaphragm. The final ILD extent score was then calculated by summing the scores of all six lung fields; the proportion of imaging abnormalities was assigned a score based on the following scale: 1 point for 1–25% involvement, 2 points for 26–50% involvement, 3 points for 51–75% involvement, and 4 points for 76–100% involvement (22, 23).

HRCT images were independently assessed in a blinded manner by two senior respiratory radiologists and one pulmonologist. Inter-observer reliability was excellent: Cohen’s kappa coefficient for visual staging was 0.89 (95% CI: 0.83–0.95, *p* < 0.001), and the intraclass correlation coefficient (ICC) for semi-quantitative scoring was 0.92 (95% CI: 0.88–0.95, *p* < 0.001). All discrepancies in scoring or staging were resolved by consensus.

### Clinical data collection

Clinical data, including general clinical information, pulmonary function parameters, and laboratory indicators (anti-cyclic citrullinated peptide antibodies (anti-CCP), rheumatoid factor (RF), serum tumor markers, and KL-6) were extracted from medical records. Additionally, serum KL-6 was quantified by latex agglutination using the Nanopia KL-6 kit (Sekisui Medical Co., Ltd., Tokyo, Japan). Other tumor markers, including carcinoembryonic antigen (CEA), CA125, CA19-9, neuron-specific enolase (NSE), and CYFRA21-1, were measured by chemiluminescence immunoassay with Cobas dedicated tumor marker kits (Roche Diagnostics GmbH, Mannheim, Germany) on a Cobas e 801 immunoassay analyzer. The normal reference ranges were as follows: KL-6 < 401.2 U/mL; CA125 < 35.00 U/mL; CA153 < 32.40 U/mL; CA19-9 < 37.00 U/mL; CEA < 5.00 ng/mL; CYFRA21-1: 0.10–3.39 ng/mL; and NSE < 10.00 ng/mL.

### Statistical analysis

Statistical analysis was conducted using IBM SPSS Statistics 27.0 and GraphPad Prism 10.0. Categorical variables were expressed as frequencies and percentages, while continuous variables were presented as mean ± standard deviation or median and interquartile range. Differences in categories were evaluated using the Mann-Whitney U test, Fisher’s exact test, the chi-square test, and the Student’s *t*-test. Forward LR logistic regression was used to identify risk factors for ILD severity in RA patients. Receiver operating characteristic (ROC) curve analysis was used to assess the sensitivity and specificity of CYFRA21-1 for predicting the severity of RA-ILD. Spearman’s correlation analysis was used to assess correlations. A two-sided *p*-value < 0.05 was considered statistically significant.

## Results

### Clinical characteristics of RA-ILD patients stratified by severity

A total of 178 RA patients were enrolled (82 without ILD, 53 with mild ILD, and 43 with advanced ILD), with an RA-ILD frequency of 53.93% (Table 1). Age, male proportion, and smoking rate increased significantly with ILD severity; the advanced group had significantly higher rates of cough and dyspnea (all *p* < 0.05). Both mild and advanced ILD groups had a lower forced vital capacity percentage of predicted value (FVC%pred) and diffusing capacity for carbon monoxide percentage of predicted value (DLCO%pred) than the no-ILD group (*q* < 0.001), and only the advanced group had elevated RF and CRP (both *p* < 0.05). DAS28-CRP, DAS28-ESR, and anti-CCP levels showed no intergroup differences (all *p* > 0.05).

**TABLE 1:** Clinical characteristics of study participants.

	RA-no ILD (*n* = 82)	RA-mild ILD (*n* = 53)	RA-advanced ILD (*n* = 43)	HC (*n* = 90)
Demographic characteristics				
Age(years)	56.06 ± 11.14	60.77 ± 9.74*	63.16 ± 8.61**	51.87 ± 13.05
Female, n(%)	64(78.05)	43(81.13)	29(67.44)	75(83.33)
Smoking, n(%)	8 (9.76)	10(18.87)	19(44.19)***	13(14.44)
Clinical manifestations				
Duration of RA(years)	6.000(3.00,8.00)	6.00(4.00,9.00)	7.00(6.00,10.00)	
Joint deformity, n(%)	32(39.02)	22(41.51)	15(34.88)	
Morning stiffness, n(%)	58(70.73)	36(67.92)	31(72.09)	
Cough, n(%)	0(0.00)	7(13.21)***	26(60.46)***	
Shortness of breath, n(%)	1(1.22)	2(3.77)	18(41.86)***	
Pulmonary function test				
FVC%pred	90.43 ± 5.75	84.92 ± 12.43*	77.57 ± 16.47***	
DlCO%pred	98.02 ± 16.39	59.30 ± 20.39***	51.94 ± 14.89***	
Indicators of disease activity				
DAS28(CRP)	4.27 ± 1.65	4.63 ± 1.91	4.21 ± 1.72	
DAS28(ESR)	4.43 ± 1.91	5.055 ± 2.08	4.72 ± 1.78	
RF, IU/mL	83.55(60.75, 153.78)	129.50(62.30, 223.40)	185.90(84.65, 371.70)***	
Anti-CCP, RU/ml	82.50(52.30, 184.30)	91.30(62.80, 175.30)	139.00(80.75, 188.70)	
Inflammatory indicators				
CRP, mg/L	27.65(12.75, 44.60)	34.50(16.20, 45.80)	37.00(24.60, 56.3)*	3.90(2.40, 9.10)
ESR, mm/h	63.80(44.488, 99.08)	62.40(52.30, 123.40)	72.50(56.35, 92.55)	12.65(5.53, 17.20)
KL-6, U/mL	224.45(178.38, 251.98)	238.30(184.80, 328.20)	1161.40(874.35, 1566.85)***	226.00(174.52, 293.17)
Tumor markers				
CEA, ng/mL	2.16(1.39, 3.31)	2.23(1.14, 3.84)	2.49(1.32, 3.68)	2.08(1.21, 3.07)
CA19-9, U/mL	18.90(12.90, 29.12)	21.70(15.60, 28.80)	41.3(14.55, 71.80)**	17.50(11.00, 26.77)
CA125, U/mL	9.10(4.88, 18.38)	10.60(5.40, 23.3)	36.20(11.35, 60.00)***	10.55(6.65, 19.25)
CYFRA21-1, ng/mL	1.90(1.33, 2.40)	2.10(1.40, 3.20)*	3.70(2.75, 4.20)***	1.70(1.23, 2.30)
NSE, ng/mL	6.45(2.90, 11.00)	9.60(4.30, 12.10)	5.10(3.44, 10.80)	5.20(3.10, 9.55)

RA-no-ILD, rheumatoid arthritis-without interstitial lung disease; RA-mild-ILD, rheumatoid arthritis-mild interstitial lung disease, RA-advanced ILD, rheumatoid arthritis-advanced interstitial lung disease, HC healthy controls, RF, rheumatoid factor; anti-CCP, anti-cyclic citrullinated peptide, FVC%pred, forced vital capacity percentage of predicted value; DLCO%pred, diffusing capacity for carbon monoxide percentage of predicted value; HRCT, high-resolution computed tomography, DAS28, 28-joint Disease Activity Score, CRP, C-reactive protein; ESR, erythrocyte sedimentation rate; KL-6, Krebs von den Lungen-6; CA125, carbohydrate antigen 125; CA19–9, carbohydrate antigen 19–9; CEA, carcinoembryonic antigen; CYFRA21–1, cytokeratin 19 fragment; NSE, neuron-specific enolase. *q < 0.05, **q < 0.01, ***q < 0.001 vs. RA-no ILD.

### Serum biomarker levels across RA-ILD severity groups

**FIGURE 1:**
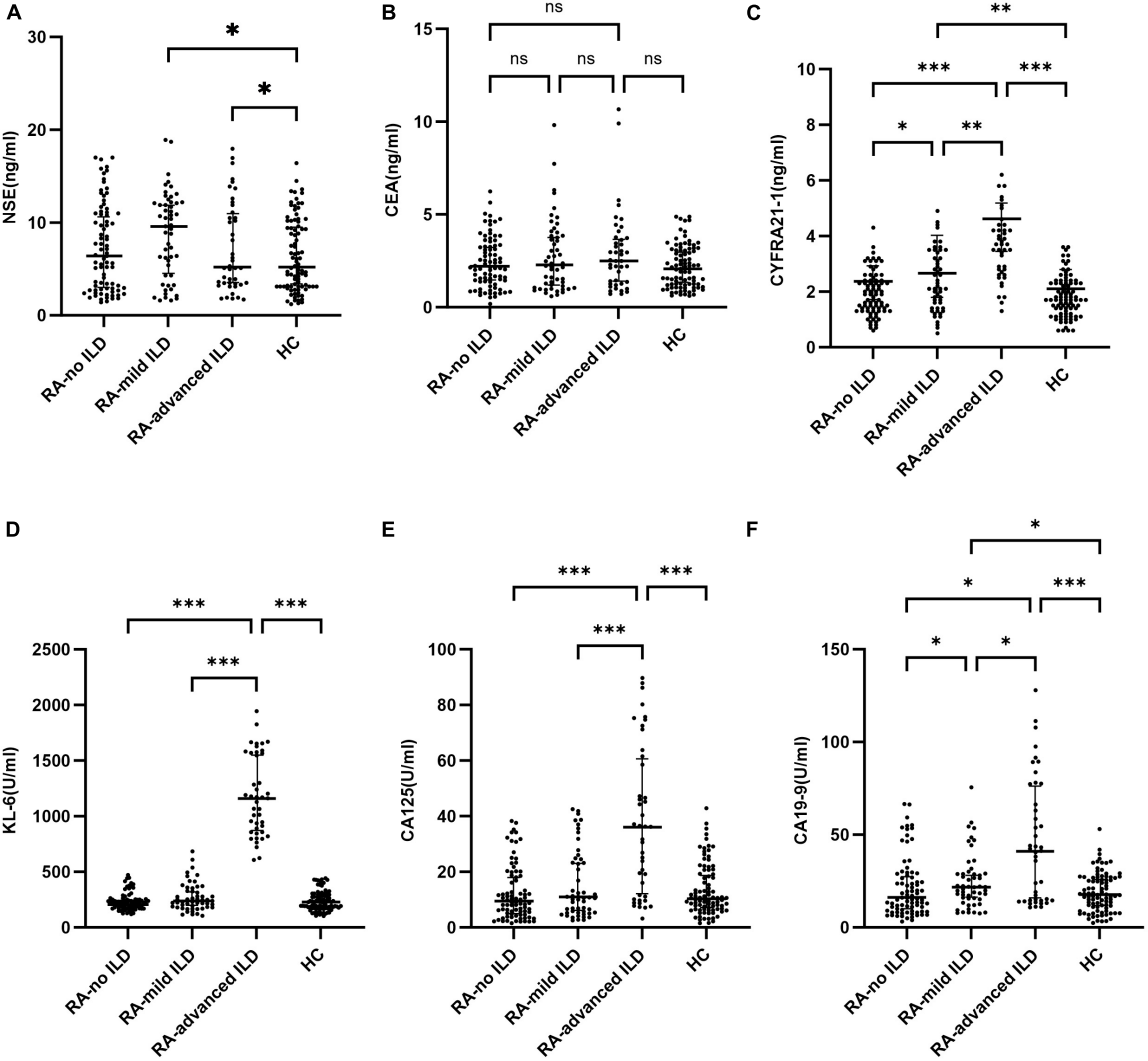
Serum levels of tumor markers and KL-6 in different groups. Serum concentrations of **(A)** KL-6, **(B)** CYFRA21-1, **(C)** CA125, **(D)** CA19-9, **(E)** CEA, and **(F)** NSE are shown as box-and-whisker plots in four cohorts: healthy controls (HC), rheumatoid arthritis without interstitial lung disease (RA-no ILD), RA with mild ILD (RA-mild ILD), and RA with advanced ILD (RA-advanced ILD). Statistical comparisons were performed using the Mann-Whitney U test with false discovery rate (FDR) correction. *q < 0.05, **q < 0.01, ***q < 0.001.

Serum biomarker levels were analyzed in all groups (Figure 1 and Table 1). CA19-9 and CA125 levels increased with RA-ILD severity, with the highest levels in the advanced group; CEA and NSE had no intergroup differences (all *p* > 0.05); moreover, CYFRA21-1 levels showed a gradient increase: 1.90 ng/mL in RA-no ILD, 2.10 ng/mL in RA-mild ILD (*q* = 0.024 vs. RA-no ILD), and 3.70 ng/mL in RA-advanced ILD (*q* < 0.001 vs. RA-no ILD). Besides, KL-6 levels in the RA-advanced ILD group reached 1,161.40 U/mL, which were significantly higher than those in the RA-no ILD group (*q* < 0.001) (Figure 1).

### Correlation analysis between biomarkers and pulmonary function

Among the tumor markers examined, only KL-6 exhibited a positive correlation with CYFRA21-1, with the correlation strength progressively increasing alongside RA-ILD severity (*r* = 0.453–0.649, all *p* < 0.05; Table 2). Both KL-6 and CYFRA21-1 showed negative correlations with FVC%pred, which also strengthened with disease severity, reaching the strongest associations in the advanced ILD group (*r* = −0.877 and −0.763, respectively, both *p* < 0.001). In contrast, CEA and NSE were only correlated with FVC%pred in the RA-no ILD group (all *p* < 0.05).

**TABLE 2:** CYFRA21-1 correlations with KL-6 and pulmonary function test results.

		KL-6	FVC% pred
CYFRA21-1 in RA-no ILD	Pearson Correlation	0.453***	−0.287**
	*P*-value	<0.001	0.009
	N	82	82
CYFRA21-1 in RA-mild ILD	Pearson correlation	0.495***	−0.768***
	*P*-value	<0.001	<0.001
	N	53	53
CYFRA21-1 in RA-advanced ILD	Pearson correlation	0.649***	−0.763***
	*P*-value	<0.001	<0.001
	N	43	43

*q < 0.05, **q < 0.01, ***q < 0.001.

### Multivariable logistic regression analysis of RA-ILD risk factors

With the RA-no ILD group as the reference, two multivariate logistic regression models were constructed using Forward LR stepwise selection (Model 1: RA-mild ILD; Model 2: RA-advanced ILD). Model 1 included only age [OR = 1.046, 95% CI (1.010–1.084)] and CYFRA21-1 [OR = 1.711, 95% CI (1.160–2.523)] as independent predictors of RA-mild ILD (Table 3, A). Model 2 indicated a significant increase in both the number and effect size of risk factors for RA-advanced ILD. Beyond age [OR = 1.074, 95% CI (1.031–1.119)] and CYFRA21-1 [OR = 4.819, 95% CI (2.942–7.892)], other factors, including smoking history [OR = 2.836, 95% CI (1.143–7.039)], RF [OR = 1.006, 95% CI (1.003–1.009)], CA19-9 [OR = 1.048, 95% CI (1.027–1.069)], and CA125 [OR = 1.085, 95% CI (1.053–1.119)] were also independent risk factors (Table 3, B). Moreover, CYFRA21-1 demonstrated a substantially stronger association with RA-advanced ILD (OR = 4.819) than with RA-mild ILD (OR = 1.711).

**TABLE 3:** Risk factors associated with different severity of RA-ILD assessed by logistic regression analysis.

Variate	OR	95%CI	*p*-value
Age	1.046	1.010–1.084	0.013
CYFRA211	1.711	1.160–2.523	0.007
**Variate**	**OR**	**95%CI**	***p*-value**
Smoking	2.836	1.143–7.039	0.025
Age	1.074	1.031–1.119	< 0.001
RF	1.006	1.003–1.009	<0.001
CA199	1.048	1.027–1.069	< 0.001
CA125	1.085	1.053–1.119	< 0.001
CYFRA211	4.819	2.942–7.892	< 0.001

(A) Mild ILD; (B) Advanced ILD. All models used RA-no ILD as the reference group. OR, odds ratio; CI, confidence interval; *p*, *p*-value.

### Diagnostic efficacy of biomarkers and combined models for RA-ILD

**FIGURE 2:**
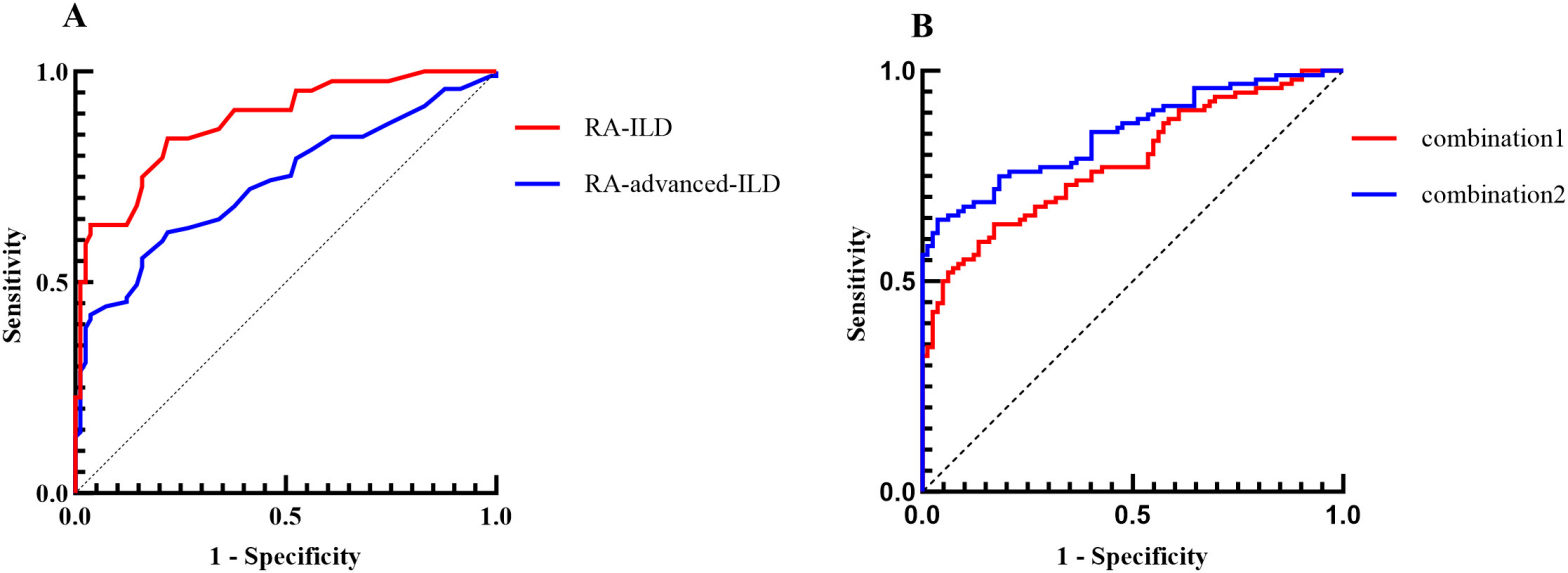
Predictive capacity of CYFRA21-1 and combined models in RA-ILD. **(A)** Predictive performance of CYFRA21-1 in RA-ILD (RA-advanced ILD + RA-mild ILD) and RA-advanced ILD. **(B)** Predictive performance of combination1 (age, RF, and CYFRA21-1) and combination2 (age, RF, CYFRA21-1, and KL-6) in RA-ILD ((RA-advanced ILD + RA-mild ILD) and RA-advanced ILD.

The ROC curve analysis evaluated the ability of CYFRA21-1 to stratify RA-ILD severity (Figure 2A). CYFRA21-1 had an AUC of 0.73 for RA-ILD (cutoff 2.45 ng/mL) and 0.88 for RA-advanced ILD (cutoff 3.53 ng/mL), with a specificity of 78.05% (all *p* < 0.001, Table 4). The combined model of age, RF, and CYFRA21-1 achieved an AUC of 0.78 for RA-ILD, and the combined model incorporating KL-6 reached an AUC of 0.85 (Figure 2B). ROC curves for other tumor markers are shown in Supplementary Figure 1.

**TABLE 4:** AUC, optimal cut-off value, sensitivity, specificity, Youden’s index, and *p*-value of CYFRA21-1 for different severity of RA-ILD by a ROC curve.

Variate	RA-no ILD vs. RA-ILD (RA-advanced ILD + RA-mild ILD)	RA-no ILD vs. RA-advanced ILD
CYFRA21-1		
Suggested cut-off	2.45	3.53
Sensitivity	61.86%	84.09%
Specificity	78.05%	78.05%
AUC	0.73	0.88
95%CI	0.66–0.81	0.82–0.94
Youden’s Index	0.3991	0.6214
*p*-value	<0.001	<0.001

## Discussion

This study demonstrates that CYFRA21-1 has superior clinical utility to traditional tumor markers in RA-ILD. These findings transcend the scope of a single diagnostic biomarker and serve as biomarkers that concurrently correlate with HRCT-assessed disease severity and pulmonary function parameters, providing crucial serological evidence for the severity stratification of RA-ILD.

Consistent with previous studies (24–30), we confirmed elevated KL-6 and CA125 in RA-ILD patients, further supporting alveolar epithelial injury as a core pathophysiological event of ILD (12, 14, 31). Although the diagnostic and prognostic value of CYFRA21-1 in IPF has been established (32, 33), the present study is the first to demonstrate that CYFRA21-1 displays a gradient expression profile in an RA-ILD cohort. This distinct expression profile, not observed for other biomarkers such as CEA and NSE, links CYFRA21-1 to irreversible pulmonary fibrosis (a severe RA-ILD feature) rather than systemic inflammation in RA, and its progressively increased OR values (from 1.711 to 4.819) in multivariate analysis further confirm its value as a severity-specific biomarker.

CYFRA21-1, a soluble fragment of cytokeratin 19 expressed in respiratory bronchioles and AECs (34), has been identified in multiple studies as an independent predictor of progressive fibrosis and poor prognosis in both idiopathic pulmonary fibrosis (IPF) and non-IPF ILDs (35, 36), serving as a potential biomarker for AEC injury in ILDs (32, 37). Its elevation likely reflects irreversible structural damage to the alveolar epithelium, such as apoptosis and EMT, rather than reversible inflammatory injury. Notably, EMT is a critical driver of pulmonary fibrosis pathogenesis and progression (38, 39).

AECs are key to alveolar barrier integrity and play a critical role in pulmonary fibrosis pathogenesis (40, 41). KL-6 reflects AEC type II stress, injury, and compensatory proliferation, serving as an early marker of lung tissue repair (42, 43). Based on the synergistic performance of KL-6 and CYFRA21-1, both showing significant negative correlations with impaired lung function, we propose an integrated correlative model of alveolar epithelial injury in RA-ILD. This model may explain the observed biomarker patterns: in the RA-mild ILD group, only CYFRA21-1 was elevated, which is consistent with epithelial remodeling processes, a characteristic pathological feature of RA-mild ILD. In the RA-advanced ILD group, both KL-6 and CYFRA21-1 showed marked elevation, which is consistent with a pathological state of ‘imbalanced injury-repair’: AEC type II undergo excessive proliferation under sustained injury, with elevated KL-6 concentrations. Concurrently, substantial epithelial cell apoptosis and structural disintegration, reflected in increased CYFRA21-1 concentrations, collectively drive extracellular matrix deposition and pulmonary architectural destruction. The simplified combined model we constructed, integrating age, RF, KL-6, and CYFRA21-1, provides an efficient and clinically feasible tool for accurate severity stratification of RA-ILD, especially for identifying RA-advanced ILD.

### Clinical translation and innovation

Anchored in alveolar epithelial injury, this study identifies KL-6 and CYFRA21-1 as pivotal biomarkers for the stratification and pathological characterization of RA-ILD, and proposes a novel, integrated biomarker strategy for its clinical management.

### Limitations and future directions

This study has several limitations. The cross-sectional design precludes inference of causality between CYFRA21-1 and fibrosis progression. The small advanced-ILD subgroup (*n* = 43) may raise overfitting concerns, warranting external validation. Furthermore, we did not adjust for treatment medications (e.g., methotrexate, biologics, corticosteroids), which could represent residual confounding. Future longitudinal studies integrating treatment data are needed to clarify the clinical utility of CYFRA21-1.

## Conclusion

In summary, CYFRA21-1 exhibits a graded expression pattern in RA-ILD and correlates positively with KL-6, with both markers showing a progressively stronger negative correlation with impaired lung function as disease severity increases. These findings support the utility of CYFRA21-1 alone and in combination with KL-6 for the accurate risk stratification and severity assessment of RA-ILD, particularly for the identification of RA-advanced ILD.

## Data Availability

The original contributions presented in the study are included in the article/Supplementary material, further inquiries can be directed to the corresponding authors.
