# Supplementary material for: Gradient elevation of serum CYFRA21-1 and its synergy with KL-6 for risk stratification in rheumatoid arthritis-associated interstitial lung disease
Source: Front Med (Lausanne). 2026 Apr 2;13:1763928. doi: 10.3389/fmed.2026.1763928 (PMC13083177; doi:10.3389/fmed.2026.1763928)
Supplement: Supplementary file 1 [file Image_1.pdf]

Supplementary Figure 1 ROC for the predictive capacity of CA125, CA19-9, and KL-6 in in RA-ILD ((RA-advanced ILD + RA-mild ILD)

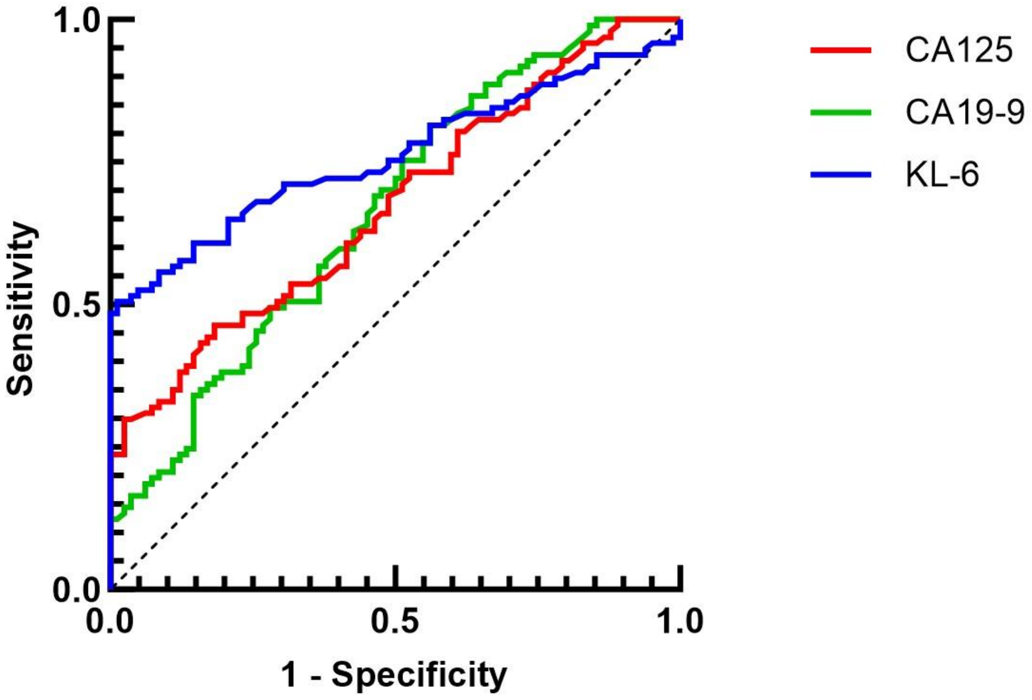

These curves depict the diagnostic predictive performance of three serum biomarkers (CA125, CA19-9, KL-6) in distinguishing patients with RA-ILD (including both RA-advanced ILD and RA-mild ILD) from the reference group (RA-no ILD). The y-axis represents sensitivity (the proportion of RA-ILD cases correctly identified), while the x-axis represents 1 - specificity (the proportion of non-RA-ILD cases incorrectly classified as positive). The dashed diagonal line denotes the reference line of random predictive chance (area under the curve [AUC] = 0.5).
